# A Light-Field-Based Method to Adjust On-Axis Rounded Leaf End MLC Position to Predict Off-Axis MLC Penumbra Region Dosimetric Performance in a Radiation Therapy Planning System

**DOI:** 10.1155/2013/461801

**Published:** 2013-10-24

**Authors:** Jia-Ming Wu, Tsair-Fwu Lee, Shyh-An Yeh, Kuan-Yin Hsiao, Hsin-Hsiung Chen, Pei-Ju Chao, Yi-Ting Chen

**Affiliations:** ^1^Department of Radiation Oncology, E-Da Hospital, Kaohsiung 824, Taiwan; ^2^Department of Medical Imaging and Radiological Science, Central Taiwan University of Science and Technology, Taichung 411, Taiwan; ^3^Medical Physics & Informatics Laboratory, Department of Electronics Engineering, National Kaohsiung University of Applied Sciences, 415 Chien Kung Road, Kaohsiung 807, Taiwan; ^4^Department of Medical Imaging and Radiological Sciences, I-Shou University, Kaohsiung 824, Taiwan

## Abstract

*Purpose*. An analytical and experimental study of split shape dose calculation correction by adjusting the position of the on-axis round leaf end position is presented. We use on-axis corrected results to predict off-axis penumbra region dosimetric performance in an intensity-modulated radiation therapy treatment planning system. *Materials and Methods*. The precise light-field edge position (*X*_tang*.p*_) was derived from the on-axis 50% dose position created by using the nominal light field for geometric and mathematical manipulation. Leaf position (*X*_mlc*.p*_) could be derived from *X*_tang*.p*_ by defining in the treatment planning system for monitor unit calculation. On-axis offset (correction) could be obtained from the position corresponding to 50% of the central axis dose minus the X_mlc*.p*_ position. The off-axis 50% dose position can then be derived from the on-axis 50% dose position. *Results*. The monitor unit calculation of the split shape using the on-axis rounded leaf end MLC penumbra region could provide an under-or overdose of 7.5% per millimeter without an offset correction. When using the on-axis rounded leaf end offset correction to predict the off-axis dose, the difference between the off- and on-axis 50% dose position is within ±1.5 mm. *Conclusions*. It is possible to achieve a dose calculation within 0.5% error for an adjusted MLC leaf edge location in the treatment planning system with careful measurement and an accurate on-axis offset correction. Dose calculations located at an off-axis spilt shape region should be used carefully due to noncorrectable errors which were found to be up to 10%.

## 1. Introduction

Multileaf collimator (MLC) systems are available on most commercial linear accelerators, for intensity-modulated radiation therapy (IMRT) treatment techniques, and many of these MLC systems utilize designs with rounded leaf ends to improve the dose profile of the geometric and transmission penumbra. The general designs of rounded leaf end MLC systems have already been described in detail by many researchers [1–8]. These MLC design considerations result in differences between the MLC 50% isodose points and the projected light-field edge locations. Before patients' treatment monitor units [9] are calculated by the treatment planning system, these differences have to be corrected. Radiation field size is defined as the lateral distance between the 50% isodose lines at a reference depth. This definition is practically achieved [10] by a procedure called beam alignment. The field-defining light is made to coincide with the 50% isodose lines of the radiation beam projected on a plane perpendicular to the beam axis and at a standard source-to-surface distance (SSD 100 cm) or source-to-axis distance (SAD 100 cm). The position of a projected split light-field edge and its relative radiation field edge of a rounded leaf end MLC needs to be measured and implemented in the computerized treatment planning system [11]. Coincidence between the 50% dose position and the split field is limited due to the nondivergent geometry found with curved leaf MLC collimator systems [12]; the 50% dose position has to be verified during MLC system acceptance. 

In order to avoid under-or overdose in patients' treatment, the treatment planning system should be calibrated precisely to 50% dose position correction when the treatment monitor units are calculated in a split MLC situation.

However, radiation dose profile measurement of leaf position is usually performed in the commissioning of the MLC system on the crosshair axis. This work will illustrate some of the specific issues that should be carefully considered if dose calculation of a split shape associated with a rounded leaf end MLC system with an off-axis setup is used [13].

## 2. Materials and Methods

**Figure 1:**
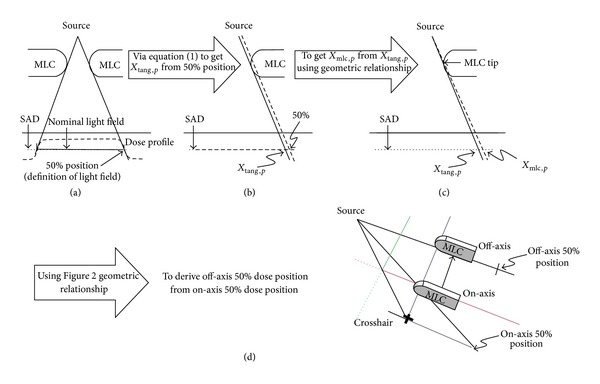
Summary of measurement procedures used in this study.

This study was performed on an Elekta Precise linear accelerator (Elekta, Stockholm, Sweden) with dual photon energies of 6 MV and 10 MV. The photon dose calculations were evaluated by using the Pinnacle v8.6 treatment planning system (Philips Healthcare, Andover, MA). Dose profiles of MLC fields were measured and the calculation results of the treatment planning system were compared for on-axis rounded leaf end MLC. The procedures of this study are given in Figure 1. 

**Figure 2:**
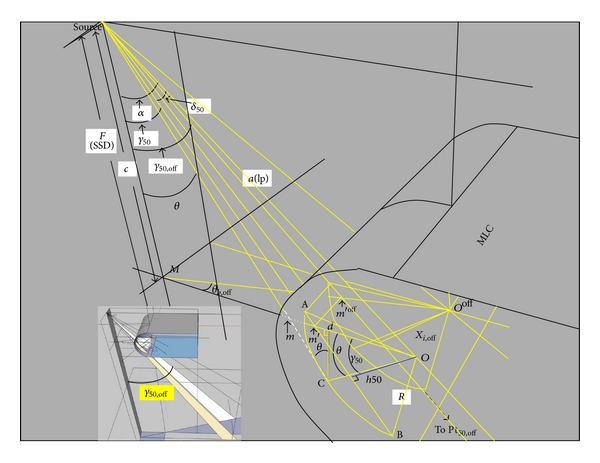
The geometric relationship for deriving the off-axis rounded leaf end MLC 50% dose position from the on-axis one.

All on-axis penumbra profiles were measured with a visual light-field (nominal light-field) at an SAD of 100 cm to determine the position receiving 50% central axis dose. The projection of the nominal light-field at SAD 100 cm was adopted for dose profile measurements, but the dose profile from the nominal light-field edge could not quantitatively determine the geometry of the tangential edge (*X*_tang,*p*_) for the derivation of *X*_mlc,*p*_ (planning system defined by leaf position); therefore, the precise light-field edge (*X*_tang,*p*_) was derived from the point corresponding to 50% of the central axis dose by geometrical and mathematical methods using (1) in this study. Leaf position (*X*_mlc,*p*_, the intersection of a line from the source to the leaf tip with SAD 100 cm plane surface in Figure 2) could then be derived from *X*_tang,*p*_. Once *X*_mlc,*p*_ was decided, the on-axis correction “offset” could be obtained by subtraction of the point corresponding to 50% of the central axis dose from the position of *X*_mlc,*p*_. The off-axis 50% dose position was then derived from the on-axis 50% dose position via the relative geometric relationship, and the off-axis offset can be predicted for comparison with the on-axis offset.

### 2.1. Geometry Specifications

All of the parameters described below are according to our previous study [14], a light-field-based method to adjust rounded leaf end MLC position for split shape dose calculation correction in a radiation therapy treatment planning system.

1. Nominal light-field.
2. *X*_tang,*p*_: light-field tangential edge position as a decimal value.
3. *X*_mlc,*p*_: treatment planning leaf position.
4. The 50% dose position.
5. Direction of the MLC.
6. Transmission penumbra.

The linear accelerators used in this study were equipped with MLCs for IMRT dose delivery devices. Many investigators have described the design and characteristics of MLCs [11, 12]. The analytical approach for optimizing the leaf design of an on-axis MLC assesses the relationship between the light-field size edge position [*X*_tang,*p*_, lp in (1)] and the 50% dose position [*X*_*i*_ or *X*_*j*_, Pt_50_(lp) in (1)].

**Figure 3:**
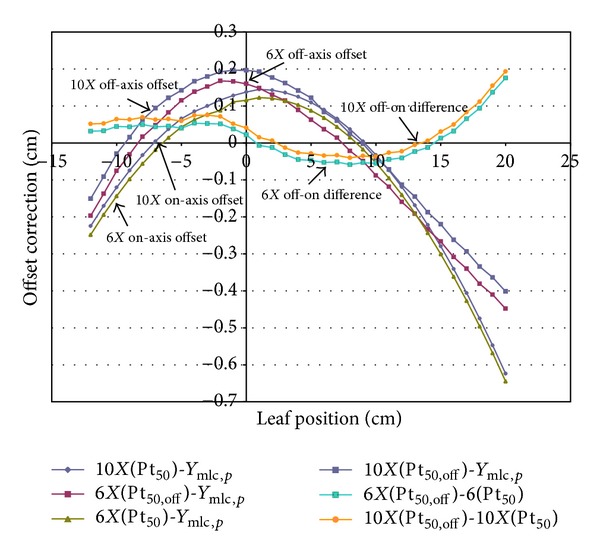
The comparison of offset correction of on- and off-axis rounded leaf end MLC.

The analytical formula of the transmission penumbra depending on leaf position will be presented in this section. In Figure 3, a schematic view of a leaf from the right bank placed at the right edge of a field is shown. If the leaf position (lp) in the field space is known, a ray line along which irradiation will drop to 50% of the initial irradiation can be defined.

By substitution of all variables in the previous study into Pt_50_(lp) = *F* · tan(*γ*_50_), the position of the point Pt_50_(lp) is given in [14]



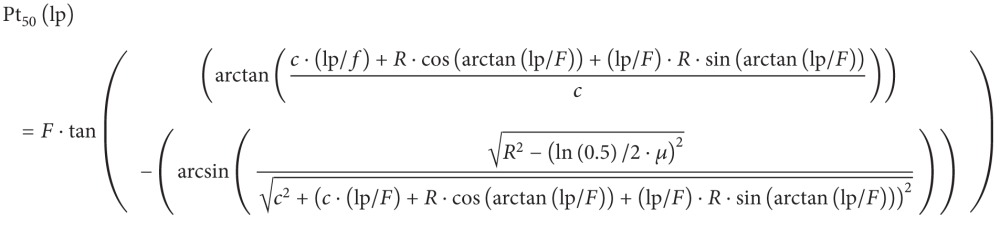
(1)

(7) Relationship of geometry and radiation position.

### 2.2. Measurement Devices

Radiation field size data was measured using water phantom scans and Gafchromic films (International Specialty Products, Wayne, NJ). A computer controlled water phantom scanning system (PTW MP3 Water Phantom Systems, PTW-Freiburg, Germany) was used for measurements with each field size. Photon diode detectors (PTW, Freiburg, Germany, p-type 60012, 1 mm active area) were used to measure the profile in air at SAD 100 cm. Measurements were also made with ion chamber (0.015 cm^3^, Type 31016 ion chambers, PTW; Freiburg, Germany, measurement point: 1.3 mm behind the chamber tip; active cylinder length: 3.6 mm; diameter: 2.9 mm) at the isocentric plane with water depths of 10 cm. The comparison of 50% dose position measured by diode in air and by chamber at water depth of 10 cm was made for the utilization of (1) in this study. All profiles were normalized on the central axis and were normalized at the centre of the irradiated area. The field sizes were defined at the 50% intensity points relative to the central value of the profile. With the same field sizes, random measurements of water phantom scans and Gafchromic film techniques were performed for the comparison of multileaf collimated field size profiles. These field sizes measured with the film agreed with the corresponding field sizes measured with water tank scans to be within 0.2 mm. After verifying that the film method achieved the same results as the water tank method, film proved to be more efficient, so we chose to use the Gafchromic film method for this study of measurements of 50% of the central axis dose.

### 2.3. Film Measurement

#### 2.3.1. Film Setup

Gafchromic film was exposed to individual rectangular fields defined by the MLC. The field sizes adopted in this experiment were generated by the nominal field size and were positioned at leaf positions from +20 cm with 1 cm increments to −12 cm (cross-over central axis −12 cm). The field size was defined at the 50% intensity points relative to the central value of the profile.

#### 2.3.2. Film Measurement and Process

The 50% dose positions were measured by Gafchromic EBT 2 film (ISP Technology, Inc., Wayne, NJ; Log F04090901; expiry date: April 2011). A double exposure technique [9–11, 15–17] was adopted for these measurements. This was performed by giving each film an initial dose of 2 Gy and measuring the optical density before experimental irradiation was applied. A variation of 2% was observed in the optical density (OD) of the films used in the experiment due to nonuniformity in the dose response [18–22].

Calibration was carried out to convert the raw scanner signal into radiation dose. This was achieved by placing 5 cm solid water phantom slabs on top of the 3.0 cm by 3.0 cm film pieces with a field size of 10 cm by 10 cm and a source-to-axis distance (SAD) of 100 cm and irradiating them with a step size of 10 cGy in the dose range from 10 cGy to 150 cGy under an Elekta Precise medical linear accelerator machine. In this study, we used an Epson Expression 10000XL flat-bed document scanner (US Epson, Long Beach, CA). Film pieces were scanned using VariSoft software (PTW, Freiburg, Germany), with the maximum OD range and all filters and image enhancement options turned off. Once the scanner is turned on, it is important to perform a preview operation in transmission mode and then to allow the scanner to warm up for half an hour. This operation turns on the upper lamp used for transmission mode and allows its temperature to stabilize. The films were scanned in the 48-bit RGB mode, with 16 bits per color, and saved as tagged image file format (TIFF) image files. 

Multiple scans are performed in order to remove the scanner noise by subsequent averaging of the scanned images. The first step in the protocol is to scan the unexposed pieces of film five times. Once the five images of the unexposed film pieces have been acquired, blank scans are taken, again five times, over the same scanning region as the previously acquired images with the film pieces. 

In general, scanned images of irradiated films will have a scanning region that is different from that of the unirradiated film pieces. Therefore, to remove the defective pixels in irradiated film images, five blank scans of the irradiated film scanning region are done. Then the processing of the images entails the identification of defective pixels. Since two glass plates are in the optical pathway, in addition to the examined films, the system can exhibit many imperfections. This identification was performed on the resulting images obtained by averaging five successive scans of the empty bed for both unirradiated and irradiated film images. We found that the percentage of faulty pixels was smaller than 0.4%.

The net OD of a point on the film is given by OD = log_10_(*S*_0_/*S*), where *S*_0_ is the background (i.e., the scanner signal for an unexposed film) and *S* is the scanner signal for the film at the point of interest.

### 2.4. Derivation of On-Axis Rounded Leaf End MLC Offset Correction

All of the parameters described below are according to our previous study [14].

1. Attenuation coefficient: *μ*.
2. Derivation of the on-axis *X*_tang,*p*_ from the on-axis 50% dose position.
3. Derivation of on-axis *X*_mlc,*p*_ from on-axis *X*_tang,*p*_.
4. The geometric derivation of *X*_mlc,*p*_.

According to our previous study [14], *∠θ* was determined from *X*_tang,*p*_ as follows:

 
*∠c* + *∠d* = 90°,
 
*∠b* + *∠c* = 90°,
 
*∠b* = *∠d*,
 
*∠d* = *∠θ*,
 
*∠a* = *∠b*,
 
*∠a* = *∠θ*,
 
$\bar{AB}=15\;\text{cm}\times \sin\theta$
 
$\left(\bar{CB}=15\;\text{cm}\right)$,
 
$\bar{AD}=15\;\text{cm}-15\;\text{cm}\times \cos\theta$,
 
$\bar{DG}=\bar{BF}-\bar{AD}$,
 
$\theta^{\prime }=\text{ta}{\text{n}}^{-1}\left(\bar{DG}/33.5\right)$,
 
*X*_mlc.*p*_ = SSD(cm) × tan(*θ*′),
  
$\bar{DE}$ = penetration thickness in MLC.
(5) Offset definition.
(6) The derivation of the off-axis 50% dose position from the on-axis 50% dose position.

To derive the off-axis 50% dose position from the on-axis 50% dose position, the previous variables for finding position Pt_50_(lp) are used again to derive the off-axis 50% dose position (Pt_50,*off*⁡_(lp)) in Figure 3.

## 3. Results

### 3.1. Film Results

According to our previous study [14], one of the film results of the split field is shown. Marker 1 on the film was 30 mm away from the crosshair isocentre and the MLC edge travelled to abut the crosshair central axis ray line for irradiation. After converting the OD to a dose distribution on the film, the position receiving 50% of the central axis dose was 31.38 mm away from marker 1 instead of 30 mm. The 1.38 mm discrepancy was due to the photon transmission and scatter effect.

### 3.2. On-Axis Offset Correction at an SAD of 100 cm

After the 50% dose position was measured from the size of the visual light-field (nominal light-field), the associated quantity of *X*_tang,*p*_ could be obtained by using (1). *X*_mlc,*p*_ could be derived from Figure 2 by implementing *X*_tang,*p*_, and the final offset corrections were calculated.

### 3.3. Off-Axis Offset Correction and the Difference between On- and Off-Axis Rounded Leaf End 50% Dose Positions

Figure 3 shows the rounded leaf end MLC uncorrectable off-axis offset correction of 10 MV and 6 MV photon beams. This figure shows three sets of curves of these two photon energies; on-axis offset correction, off-axis offset correction, and the difference of 50% dose positions between off- and on-axis rounded leaf end MLC at an SAD of 100 cm.

## 4. Discussion

In order to simplify the model for the transmission penumbra, the source was here approximated by a point. The coincidences of the 50% dose position measured by diode in air and the chamber with water depth 10 cm in water phantom support the utilization of transmission penumbra model in (1) in this study. The precise leaf edge position of the tangential split field (*X*_tang,*p*_) could be derived using the measured on-axis 50% dose position from the mathematical model and can be used to obtain the planning system defined by leaf position (*X*_mlc,*p*_). The on-axis offset (the 50% dose position minus the planned leaf position) could be determined for the purpose of accurate monitor unit calculation. If the MLC rounded leaf travels close to the central axis, the 50% dose position gains attenuation and will be projected outside *X*_mlc,*p*_ on *X*_*j*_. As the MLC rounded leaf travels away from the central axis, the 50% dose position will be projected inside *X*_mlc,*p*_ and gain less attenuation, as shown by *X*_*i*_. This offset adjustment can be of importance in clinical situations of split fields to determine overdosage or underdosage at treatment of SAD.

*X*_mlc,*p*_ was calculated by a mathematic analytical model at (1). According to our previous study [14], it shows one of the films in the experimental setup along with the profile result of the split light-field edge and the position receiving 50% of the central axis dose at an SAD of 100 cm with a 10 MV photon beam. Marker 1 was delineated by the jaw edge 30 mm from the crosshair isocenter, and marker 2 (used for double-checking the position setting accuracy) was 15 mm away from the centre of marker 1. Fifty percent of the central axis dose can be found via the profile through an OD-to-dose conversion; this moves away from the central axis toward the MLC shadow due to side scattering of photons and electron contributions.

This result of film measurement showed the positions of the 80% dose and the 20% dose at 26 mm and 34 mm, respectively. The width of the split field penumbra from the 80% dose to the 20% dose was approximately 8 mm and changed with the rate of dose gradient by 7.5% per mm at an SAD of 100 cm with a 10 MV photon beam. Monitor unit calculation in the treatment planning system is decided entirely by the selected point on the split field penumbra curve. When the point is selected on the descending or ascending portion between the 50% dose and the 20% dose, or between the 80% dose and the 50% dose, the results for monitor units will be over- or undercalculated.

The 50% dose position was larger at 10 MV than at 6 MV because photons have greater penetration at 10 MV. When patients treatment monitor units are calculated in a split field situation, the on-axis offset (50% dose position minus *X*_mlc,*p*_) correction should be calibrated precisely to avoid underdosage or overdosage of patients. The calculated monitor units for treatment will be less than the desired dose and lead to under-dosage due to overcorrection because the point receiving 50% of the central axis dose used for monitor unit calculation passes through the ascending portion from 50% to 80%. The 50% point used for monitor unit calculation passes through the descending portion from 50% to 20%, so the underestimated output selected in this region will lead to overcalculated monitor units and will result in over-dosage. The 50% dose position was located outside *X*_mlc,*p*_ (away from the source), since more attenuation leads to the positive offset correction in the range from +8 cm to −8 cm, whereas the negative offset correction is in the range from −12 cm to −8 cm and from +20 cm to +8 cm because the 50% dose position is located inside *X*_mlc,*p*_ (close to the source).

We expand the rounded leaf end right and left sides to simulate the off-axis MLC interaction with photon beams when leaf is at off-axis setting. Figure 3 shows the results of off-axis offset when leaf is at the leaf tip level of 6.3745 cm off-axis location (*X*_*i*,*off*⁡_ = 6.374 cm). The *X*_*i*,*off*⁡_ with a ±6.3745 cm off-axis distance at the leaf tip level (*c* distance level) has the extreme light-field size projection of ±20 cm at SAD 100 cm plane (since leaf width is 1 cm at SAD 100 cm, *c* is 33.55 cm). This study not only shows that the 50% dose position created by the projection of MLC split edge shifts away from the central axis towards MLC shadow, but also demonstrates the same photon scatter phenomenon at off-axis distal MLC positions. The off-axis offset of the 50% dose position is located much further outside than the on-axis position when the leaf position is larger than 15 cm. This deficient tangential attenuation (off-axis 50% dose position minus on-axis 50% dose position) leads to a trend of positive curves in the upper right part of Figure 3.

Table 1 shows how to derive the off-axis 50% dose position from the on-axis 50% dose position of rounded leaf end MLC of 10 MV and 6 MV photon beams at an SAD of 100 cm.

The 50% dose position of off-axis 50% rounded leaf end MLC could be derived from the ratio of *Mm* ^′*off*⁡^ (column 6 in Table 1) and *MO* ^*off*⁡^ (column 5 in Table 1) to that of *Mm*′ (column 3 in Table 1) and *MO* (column 4 in Table 1). The angle *γ*_50,*off*⁡_ is then tan^−1^(*Mm* ^′*off*⁡^/c) (column 7 in Table 1), and therefore Pt_50,*off*⁡_ (column 8 in Table 1) is calculated by *F* · tan⁡(*γ*_50,*off*⁡_). The identification field size (visualized field size of Pt_50,*off*⁡_ projection on MLC moving direction) of off-axis rounded leaf end MLC is calculated by Pt_50,*off*⁡_ · cos⁡(*θ*_*b*,*off*⁡_) (column 10 and 11 in Table 1).

We set lp to be intentional from −12 cm to −11.85 cm to simulate leaf position to be in 1.5 mm error intentionally, as a result for 6 MV and 10 MV offset correction with value from −0.31337 cm to −0.26369 cm, and from −0.32337 cm to −0.27369 cm, respectively. The offset correction differences of 6 MV and 10 MV are around 0.5 cm (0.31337 cm–0.26369 cm or 0.32337 cm–0.27369 cm). The rate of dose gradient is around 7.5% per mm at an SAD of 100 cm with 10 MV and 6 MV photon beams; therefor we adopt an action level for leaf position adjustment while leaf position error is larger than 1.5 mm, because this error leads to a dose calculation error around 3.5% (7.5% divided by 2).

## 5. Conclusions

It is critical for high-quality radiation therapy that planned and delivered dose measurements should be at an appropriate level. In this study, we illustrate that the accumulated and planned radiation doses may not always be in agreement for MLC treatment fields at an SAD unless the offset is carefully adjusted.

With careful measurement and an accurate on-axis offset correction, it is possible to achieve dose calculation within 1.0% error for the adjusted MLC leaf edge location on-axis in the treatment planning system.

Calibration could be performed at a certain on-axis SAD to fit all off-axis offset corrections. We should keep in mind that patient treatment monitor unit calculations at extremely off-axis settings could result in significant uncorrectable underdosage or overdosage in treatment planning dose calculation. 

## Figures and Tables

**Table 1:** This table demonstrates the derivation procedures of off-axis 50% dose position from on-axis 50% dose position of 10 MV and 6 MV photon beams at an SAD of 100 cm.

1	2	3	4	5	6	7	8	9	10	11
Nominal field size	*γ*_50_ of nominal field	*Mm*′ of on-axis	*MO* of on-axis	*MO* ^*off*⁡^	*Mm* ^′*off*⁡^	*γ*_ 50,off_	Pt_50,off_	Θ_*b*,*off*⁡_	10*X*, Pt_50%,off_ · cos⁡(θ_*b*,*off*⁡_)	6*X*, Pt_50%,off_ · cos⁡(θ_*b*,*off*⁡_)
−12	−0.119	−4.018	10.081	10.625	−4.235	−0.126	−12.623	0.321	−11.977	−11.797
−11	−0.109	−3.683	10.398	10.926	−3.870	−0.115	−11.535	0.312	−10.977	−10.807
−10	−0.099	−3.347	10.719	11.232	−3.508	−0.104	−10.455	0.303	−9.977	−9.808
−9	−0.090	−3.012	11.038	11.537	−3.148	−0.094	−9.383	0.295	−8.978	−8.818
−6	−0.060	−2.006	12.010	12.470	−2.082	−0.062	−6.207	0.272	−5.978	−5.830
−5	−0.050	−1.670	12.336	12.784	−1.731	−0.052	−5.159	0.266	−4.978	−4.836
−4	−0.040	−1.335	12.669	13.106	−1.381	−0.041	−4.115	0.259	−3.978	−3.826
−3	−0.030	−0.999	12.999	13.425	−1.032	−0.031	−3.076	0.253	−2.978	−2.830
−2	−0.020	−0.664	13.332	13.748	−0.684	−0.020	−2.040	0.247	−1.978	−1.826
−1	−0.010	−0.328	13.663	14.069	−0.338	−0.010	−1.007	0.241	−0.978	−0.835
0	0.000	0.007	13.995	14.391	0.007	0.000	0.022	0.235	0.022	0.156
1	0.010	0.343	14.325	14.712	0.352	0.010	1.049	0.230	1.021	1.137
2	0.020	0.678	14.662	15.041	0.696	0.021	2.074	0.225	2.021	2.135
3	0.030	1.014	14.996	15.367	1.039	0.031	3.096	0.220	3.021	3.120
4	0.040	1.349	15.333	15.696	1.381	0.041	4.116	0.215	4.021	4.109
5	0.050	1.685	15.674	16.030	1.723	0.051	5.135	0.211	5.021	5.109
6	0.060	2.020	16.016	16.364	2.064	0.061	6.152	0.206	6.021	6.103
7	0.070	2.356	16.360	16.701	2.405	0.072	7.167	0.202	7.021	7.103
8	0.080	2.691	16.703	17.036	2.745	0.082	8.181	0.198	8.021	8.092
9	0.090	3.026	17.050	17.377	3.085	0.092	9.194	0.194	9.021	9.091
10	0.100	3.362	17.395	17.716	3.424	0.102	10.205	0.191	10.021	10.081
11	0.110	3.697	17.745	18.060	3.763	0.112	11.216	0.187	11.021	11.080
12	0.120	4.033	18.093	18.401	4.102	0.122	12.226	0.183	12.021	12.070
13	0.129	4.368	18.446	18.748	4.440	0.132	13.234	0.180	13.021	13.069
14	0.139	4.704	18.796	19.093	4.778	0.141	14.242	0.177	14.021	14.059
15	0.149	5.039	19.152	19.443	5.116	0.151	15.249	0.173	15.021	15.058
16	0.159	5.375	19.505	19.791	5.454	0.161	16.256	0.170	16.020	16.048
17	0.169	5.710	19.863	20.144	5.791	0.171	17.262	0.167	17.020	17.047
18	0.178	6.046	20.219	20.495	6.129	0.181	18.267	0.164	18.020	18.037
19	0.188	6.381	20.579	20.851	6.466	0.190	19.271	0.162	19.020	19.036
20	0.198	6.717	20.938	21.205	6.802	0.200	20.276	0.159	20.020	20.025
